# New Arrangement

**Published:** 1859-02

**Authors:** 


### NEW ARRANGEMENT.

By reference to the Advertisement of the next Regular Course of Lectures in the Atlanta Medical College, it will be perceived that General Pathology has been transferred to the Chair of Practical Medicine, where it appropriately belongs, and Diseases of Women and Children, have been added to the chair of Physiology. Obstetrics alone furnishing a more than sufficient field for the Professor of that Department to occupy, in the limited time allowed, and the Diseases of Women and Children, each, having grown up to be a science, it has been thought that more prominence (by extra and special lectures) given to these subjects, would add to the value of the Course of Instruction.

The connection with the department of Physiology, seems to be peculiarly appropriate, as the Professor of that science must necessarily direct particular attention to the peculiarities of the female and infantile organizations.

The subjects of Reproduction, Menstruation, and the Fœtal Circulation, along with other Physiological peculiarities in both mother and offspring—constituting essentially, a *Female* and an *Infantile Physiology*—belong to the Chairs of Obstetrics and Physiology in common, and it is thought may be made an exceeding interestingly and valuable addition to the latter.

It will thus be perceived in this, as in every arrangement of the Course of Instruction, constant reference is had to *practical* teaching, and the intimate connection of the *beauties* and *uses* of the science.

